# ‘We Should Not Be Quiet but We Should Talk’: Qualitative Accounts of Community-Based Communication of HIV Pre-Exposure Prophylaxis

**DOI:** 10.1177/10497323231181207

**Published:** 2023-07-05

**Authors:** Hannah Goymann, Mxolisi Mavuso, Shannon A. McMahon, Anita Hettema, Allison B. Hughey, Sindy Matse, Phiwa Dlamini, Kathleen Kahn, Till Bärnighausen, Albrecht Jahn, Kate Bärnighausen

**Affiliations:** 1 Institute of Global Health, 9144 Heidelberg University, Heidelberg, Germany; 2 Schools Health Services, Mbabane, Eswatini; 3 384927 Clinton Health Access Initiative, Mbabane, Eswatini; 4 299373 Ministry of Health, Mbabane, Eswatini; 5 MRC/Wits Rural Public Health and Health Transitions Research Unit, School of Public Health, 37707 University of the Witwatersrand, Johannesburg, South Africa; 6 Department of Global Health and Population, 1857 Harvard T.H. Chan School of Public Health, Boston, MA, USA; 7 560159 Africa Health Research Institute, Kwazulu-Natal, South Africa; 8 School of Public Health, 37707 University of the Witwatersrand, Johannesburg, South Africa

**Keywords:** community leaders, PrEP, Eswatini, community-based communication, HIV prevention, qualitative research

## Abstract

Community leaders play an important role in the acceptance of public health services, but little is known about their willingness to facilitate HIV pre-exposure prophylaxis (PrEP) roll-out in Eswatini. We conducted in-depth interviews (*n* = 25) with purposefully selected male and female community leaders in Eswatini. We analysed our data inductively using a thematic analysis approach. Community leaders feel they are important communicators of culturally appropriate PrEP messaging. Our participants described a complex social space within their communities influenced by religion, tradition, values, and HIV stigma. Community leaders use their position to provide leverage for unique, effective, and easily accessible messages and platforms to reach the community in a manner that ensures trust, relatability, familiarity, and shared faith. Community leaders feel that they are trusted and see trust manifesting in the conversations they are able to engage in, and have a reach that extends beyond formal health services. Existing PrEP programming should embed community leader participation in PrEP programming and engage the trust, knowledge, and potential of community leaders to support PrEP uptake and acceptance.

## Introduction

Community leader (CL) participation in HIV prevention programs has been shown to be culturally appropriate (Carm, 2012), context-relevant (Bulled & Green, 2016; Campbell, 2010), and responsive to the urgent need to reduce the incidence of HIV in rural communities, particularly in sub-Saharan Africa (Chimatiro et al., 2020). While community-based programs – tailored to local and cultural specific needs of the target population (Gautier et al., 2020; Mulumba et al., 2018) and owned by the community – can support the local health system, are sustainable (Decroo et al., 2013), and can effectively decentralise health care (Mukherjee et al., 2016), little is known about CLs involvement in the implementation of pre-exposure prophylaxis (PrEP) for HIV prevention, an important prevention function of health systems in communities hard hit by the HIV pandemic.

CLs play an important role in the community-based implementation and promotion of health programs (Stankevitz et al., 2019) and behavioural change (Tarkang et al., 2018), due to their understanding of how health programs are viewed and received within their cultural context (Tarkang et al., 2018). CLs are often perceived as role models, can disseminate health information, offer appropriate community platforms for external health agencies, and support programs after external agencies have withdrawn (Valente & Pumpuang, 2007).

For HIV prevention, this unique reach that embodies culturally appropriate and context-specific messaging is particularly important due to the social complexities and phenomena that have arisen from HIV and AIDS (Root, 2014). Culture is defined as a complex and dynamic concept that “*is learned, shared, transmitted intergenerationally, and reflected in a group’s values, beliefs, norms, practices, patterns of communication, familial roles, and other social regularities*” (Kreuter & McClure, 2004 p. 440). Much stigma, for example, stems from the fear of HIV combined with campaigns or strategies that came from culturally inappropriate internal and external sources and perpetuated narratives around the sexual deviance of disenfranchised groups, further marginalising those living with HIV and vulnerable to HIV infection (Campbell, 2005; Nyblade et al., 2009). CLs are thought to bypass much of this and bring the realities of their communities to the forefront of their approach to providing HIV prevention information and care (Carm, 2012). Because of this, Carm (2012) demonstrates that CLs can serve as crucial gatekeepers, change agents, and local capacity builders for HIV prevention and recommends their active involvement in HIV prevention programs (Carm, 2012).

Although shown to effectively reduce HIV transmission (Donnell et al., 2014) and endorsed by the World Health Organization (WHO) as a prevention method for people at high risk of HIV acquisition (World Health Organization et al., 2015), HIV PrEP uptake and adherence – especially in high incidence rural communities – has been suboptimal (Geldsetzer et al., 2020; Koss et al., 2020). Low-risk perception (Camlin et al., 2020), demand for more information (Camlin et al., 2020; Geldsetzer et al., 2020), fear of being stigmatised (Camlin et al., 2020; Geldsetzer et al., 2020), difficulties adhering to an oral daily routine (Camlin et al., 2020; Geldsetzer et al., 2020), side effects (Camlin et al., 2020; Geldsetzer et al., 2020), and structural barriers (Camlin et al., 2020; Irungu & Baeten, 2020) are issues attributed to lower than expected uptake. Several approaches are now being trialed to increase the uptake and use of PrEP (Morton et al., 2020) by those who could benefit from its use the most but, to the best of our knowledge, none consider actively involving CLs in PrEP programs in sub-Saharan Africa (Young et al., 2021).

In Eswatini, PrEP is available for the general population via public and private clinics (PrEP Demonstration Study in Swaziland, 2017), but despite the deeply embedded, respected, and influential community leadership system, the opinions and involvement of CLs in PrEP implementation are absent from implementation guidelines and policy. Recent qualitative studies from Eswatini call for PrEP promotion and education in community settings (Berner-Rodoreda et al., 2020; Geldsetzer et al., 2020) and to involve CLs in the promotion and implementation of PrEP (Bärnighausen et al., 2020).

Our qualitative study stems from formative research conducted within the Eswatini PrEP demonstration project where data collected with PrEP clients, stakeholders, and health workers highlighted CLs as central to PrEP demand creation, awareness, and promotion. This research (Bärnighausen et al., 2020) states that the most common themes throughout the dataset were the need for CL participation, community-based engagement, and community-led demand creation (Bärnighausen et al., 2020).

Therefore, we explore the perspectives of CLs and their role in the promotion of PrEP in their community. We describe the CLs’ suggestions and perceived challenges regarding PrEP and highlight where CLs can be used to assist to disseminate information about PrEP and better support broader PrEP interventions within their respective communities.

## Methods

### Study Setting

The Kingdom of Eswatini is a landlocked country with borders to Mozambique and South Africa (United Nations, 2019). With its dual governmental structure, Eswatini is ruled by a dual system of traditional and constitutional institutions (Government of Eswatini, 2021) which are equally important in policy development and legal practices. Our study was conducted in rural and semi-urban sites within the Hhohho region, the administrative capital of Eswatini. In 2017, Eswatini’s population was 1.1 million, with 79% of people living in rural areas (United Nations Eswatini, 2020). Eswatini has reached the UNAIDS 95-95-95 targets (UNAIDS, 2021a) but still – at approximately 27% – has the highest HIV/AIDS prevalence worldwide. 90% of Eswatini’s population identifies as Christians (Bertelsmann Foundation, 2020). Traditional values and beliefs play an important role in the structure and legislation of Eswatini’s society (Ndlovu, 2015).

Ndlovu (2015) explains how Eswatini culture is deeply embedded across society, from the individual to the respected community system, with the polygamous king and his mother being powerful representatives of national culture and identity (Berner-Rodoreda et al., 2023). Eswatini’s local governmental system is divided into 55 Tinkhundla and 385 chiefdoms, organised in different Tinkhundla, which form local, rural authorities and aim to work on a local community level (The Government of The Kingdom of Eswatini, 2021) (Dlamini, 2013). Tinkhundla (many) or Inkhundla (one) is a place where the community meets to discuss local issues, make announcements, or elect CLs.

An Inkhundla usually has many communities under it and each community has its centre to meet and discuss problems of that particular community. These centres are called Umphakatsi (one) and Imiphakatsi (many). In every chiefdom, CLs, called Bucopho, are elected to represent the chiefdom within the Inkhundla and are responsible for the care and development of their community. Bucopho, chiefdoms, and Tinkhundla are the link between community and government. Authorities within the local government are gatekeepers to the communities and decide the agendas of importance to be discussed at the local level (Dlamini, 2013).

### Study Design

We employed a case study design and conducted semi-structured in-depth interviews (IDIs) with CLs working within the catchment area of the PrEP demonstration project in the Hhohho region in Eswatini. We purposively selected the CLs to capture diversity in age, gender, and position within the community. CLs from rural and semi-urban areas were identified through the community leader system within their respective chiefdoms.

### Data Collection

We purposively selected 25 participants from similar areas where the demonstration project had taken place. Participants were interviewed once by local research assistants (RAs) fluent in SiSwati and English. RAs (*n* = 1 male and *n* = 1 female) were proficient in qualitative data collection and received additional qualitative data collection training from the study leads. After obtaining written informed consent, the interviews were conducted one-on-one in a private undisturbed location, chosen by the participant, and audio recorded. Any personal information was anonymised. Interviews lasted between 22 to 66 minutes, were conducted in SiSwati, and captured socio-demographic characteristics such as age, sex, education, and employment status. Participants were asked broad, open-ended questions about the challenges of HIV prevention in communities and the role CLs play in health education while focusing on PrEP and its implementation within their communities.

Reflexive and observational notes were made during and after interviews and interviews were transcribed and translated into English verbatim, and several transcripts were randomly selected for quality checks by an external party. We conducted daily debriefings to facilitate reflexive discussion, to discuss the content of the interviews, and to make amendments to the interview guide if required (McMahon & Winch, 2018). We also used these debriefing sessions to make an initial decision as to whether we were reaching data saturation (Fusch & Ness, 2015). After approximately five interviews, we – alongside the other debriefing questions – asked whether the interview was rich in description, whether they were hearing similar answers, and if any new insights were coming from the interviews. Given our sample specificity and the quality of dialogue that our RAs reported, at approximately 20 interviews, we felt we had enough ‘information power’ (Malterud et al., 2016) and stopped data collection at the 25^th^ participant.

### Data Analysis

Data were analysed using an inductive approach of thematic analysis (Braun & Clarke, 2006). Analysis of transcripts, debriefings, and reflexive and observational notes was conducted by the first author, who was not part of the data collection team, and by the last author, who led data collection.

The first phase of analysis involved reading and rereading the transcripts and making notes to achieve data familiarisation. The first and last authors discussed initial thoughts, and then data were coded and organised into main themes. After reviewing and discussing the different focuses of the main themes, we conducted another round of coding of all transcripts, focusing on allocating codes to the most prominent themes. We felt our themes could be structured to describe the social context of rural Eswatini, but also resonated with much communication theory. We reorganised the codes into 17 basic themes and clustered them into four main organising themes: the social context, the message content, the message delivery, and the message persuasion, which also serve as our organising sub-headings for the results section.

To refine the basic themes and develop the final codebook, the first author went back to the dataset until all codes were allocated to the basic themes (Table 1).

To minimise analysis bias (Smith & Noble, 2014), the first author did not conduct any literature based research until completing the first round of coding. Due to the inductive approach of this analysis, the themes were not informed by any predefined coding scheme, and interim results were discussed and compared regularly between two researchers (the first and last author). Data were managed using NVivo Pro 12 (Castleberry, 2014).

**Table 1.** Coding Framework.

Codes	Basic themes	Organising themes
- HIV education in church	- Religious spaces	**Social context**
- HIV education of men	- Traditional values	
- HIV education of the youth	- Individual values	
- HIV education of women	- Stigma	
- HIV education of elders		
- Parents teaching their children		
- Lack of information and misunderstanding		
- People do not want to get tested		
- Impact of culture on HIV		
- Role of the church in HIV issues		
- Stigma in Eswatini		
- Critical points related to the implementation of PrEP		
- Invite NGOs to talk about HIV	- HIV prevention	**Message content**
- Consumption of alcohol leads to HIV	- Empowerment	
- Marula season increases alcohol consumption	- Risk factors	
- People feel embarrassed to buy condoms	- Referrals	
- Female empowerment	- Stigma reduction	
- Can CL talk about HIV openly with the community		
- Promotion of condom use to prevent HIV		
- What do you do if someone asks you about PrEP?		
- CLs and health education		
- Stigma in Eswatini		
- Is it difficult to talk about HIV with different gender?	- One-on-one communication	**Message delivery**
- CLs cooperating with health NGOs	- Communication with larger groups	
- Can CLs talk about HIV openly with the community?	- Church as important communication platform	
- CLs talking to different age groups		
- How would you (CL) advice someone to use PrEP?		
- Methods proposed to inform the community about HIV in general		
- Methods proposed by the CLs to implement PrEP		
- Happy about PrEP as a new prevention method	- Trust	**Message persuasion**
- Where should PrEP and information about PrEP be offered	- Knowledge	
- Can CLs talk about HIV openly with the community?	- Identification	
- CLs duties in the community	- Availability	
- CLs health duties in the community	- Empathy	
- CLs working in the community with health issues		
- Knowledge about HIV		
- Knowledge about PrEP		
- Methods proposed by the CLs to implement PrEP		
- Methods proposed to inform the community about HIV in general		
- CLs are motivated to promote PrEP		
- CLs and health education		
- Recommendations to educate men about HIV prevention		
- Did the CLs receive training on HIV prevention?		
- Would you (CL) like to receive a training on PrEP?		
- Would CL support PrEP?		
- Community trusts the CLs		
- Do people listen to you as a CL?		
- Impact of religion		
- Impact of traditional healers		
- CLs living with HIV		

### Ethics

The trial was approved by the Eswatini Ministry of Health National Health Research Review Board (MH/599C/IRB 0009688/NHRRB538/17) and the USA Chesapeake Institutional Review Board (Pro00021864), and exempted by the Heidelberg Ethics Commission.

## Results

We conducted in-depth interviews with 11 female and 14 male CLs from rural and urban sites in the Hhohho region of Eswatini. The majority (*n* = 16) of the interviewed CLs were between 20–49 years old and had completed High School. Ten of the interviewed CLs were Bucophos and the remaining 15 obtained different leadership positions within the community. Seven of the interviewed CLs had not received any training on HIV so far and 18 had taken part in at least one training event. Almost half of the interviewed CLs had been leaders for 1–10 years. Four CLs had held their position for 15 years, whereas nine CLs had been CL for less than one year (Table 2).

**Table 2.** Respondent Characteristics.

Community leaders		
	N	%
Gender		
Female	11	44
Male	14	56
Age, years		
20–29	1	4
30–39	6	24
40–49	9	36
50–59	2	8
60–69	6	24
70–79	1	4
Missing	0	0
Position		
Bucopho	10	40
Indvuna	4	16
ye Nkhundla		
Chiefdom	2	8
Other	9	36
Missing	0	0
Period in charge, years		
<1	9	36
1–10	12	48
15	4	16
Level of education		
Primary education	4	16
High School	16	64
Tertiary education	3	12
Vocationally training	1	4
Missing	1	4
HIV training received		
Yes	18	72
No	7	28

We identified CLs, along with traditional healers, and religious leaders as important communicators and promotors of effective, culturally appropriate communication of HIV prevention and PrEP programs for communities in Eswatini. First, we present the context in which CLs operate, which is a complex space that affects the content and delivery of HIV and PrEP information. We then present our results in line with our organising themes which describe the message content, the message delivery, and the message persuasion techniques employed by the CLs. This roughly aligns with ‘the who, the what, and the how’ of Lasswell´s (1948) description of communication as an interaction of “*Who, Says What, In Which Channel, To Whom, With What Effect?*” (Kreuter & McClure, 2004; Lasswell, 1948; p. 216).

### Community Context

CLs describe a unique and complex space within their communities steeped in the conflict between HIV prevention and religion, culture, and values. Stigma and misinformation are commonplace, and men and young people are seen as particularly hard to reach. The CLs considered HIV a main challenge for the health of Eswatini and can see the need for HIV education, want their communities to be educated and informed about PrEP and are highly motivated to support the implementation of PrEP. CLs explained they “want to be Bucopho to a healthy community” (63 y, m).

The CLs described a space within their communities where religion is the dominating influence on social and health behaviour. One CL described religion and its influence on HIV as “a very difficult and hot one [issue] to handle. We are now talking of a big issue. Jesus” (71 y, m). The CLs felt that some religious beliefs do present a conflict between the open promotion of HIV prevention and the need to provide information about HIV, since the church members think that “when you talk about sexual matters people think you are no longer a Christian” (50 y, f). If addressing issues on sexuality and HIV prevention, the community member “will say that my religion does not allow me to talk about these things” (63 y, m).

This space is further mediated by traditional beliefs passed on via parents and grandparents. CLs explained that – in a context where much of the older generation was lost (taking their history with them) and approximately a third of all children were orphaned by AIDS – this is significant because of the status and role of elders within families and the need to guard what they hold as important, and to protect and preserve Eswatini traditions. Traditions and traditional beliefs passed down by elders represent ‘real’ Eswatini culture, and the weight and influence of these people could not be underestimated.

This was highlighted by one example, given by a male CL (35 y): “The girl would say: my mother said I should not really listen to what you say [about HIV] because she [the mother] was taught by her grandparents about these things”. Young adults “concentrate on what their parents taught them, even if they do want to listen to you because their parents insist that they are more knowledgeable” (35 y, f). However, some CLs noted that these traditional values can also be quite conservative, and some community members prefer that their children are not spoken to about sex since they fear that people will “teach the child bad habits about sex” (35 y, m).

Further embedded within the traditional beliefs, traditional medicine, promoted by traditional healers, plays an important role in the communities’ health behaviour. Traditional medicine is a frequently used and trusted HIV prevention and treatment option. One CL told us that after visiting a traditional healer, some people stop taking their medical treatment, “even if you were taking antiretroviral therapy (ART) they stop” (66 y, f), and a CL explained that as a health worker alone you “can’t change their traditional ways of culture” (33 y, m).

Men were described as the group that was more likely to use traditional medicine because the “men are reliant on traditional medicine, they believe that *bayaloywa* [they are bewitched], they are heavily reliant on concoctions and once they get sick with HIV they will never know because they refuse to get tested, they will tell you that someone is bewitching them so what happens next they will go up and down to different traditional doctors or healers to get more concoctions just to fight the thing that they have which is HIV” (71 y, m). One female CL told us that “they [the men] are scared” (63 y, f) to go to the hospital and prefer visiting their traditional healer if they feel sick. Even for her husband, one CL explained, that “when he is sick, we look for herbs to heal him” (57 y, f).

Men were also described as a group that is very hard to reach and talk to about HIV and will only go and seek help if they are in a very bad health condition, “being pushed in a wheelbarrow” (39 y, m). The CLs felt that men are afraid that if “they go and learn [about HIV prevention] they will be seen as someone that wants to be promiscuous” (49 y, m). Moreover, some men fear that “after testing you will never be able to have kids” (71 y, m). They “hate lining up” (71 y, m), and the procedure in the hospitals “just annoys them” (48 y, f). Men think that HIV does not affect them and “they think it is beneath them to learn about this [HIV]. They think it is silly for them to learn” (35 y, m). Besides, “men have got this tendency of saying let the woman go” (48 y, f) to the hospital and “test the waters for them” (48 y, f). They believe that HIV is “a woman’s thing” (29 y, m), and if their wife tests negative for HIV, they are as well. Moreover, the CLs told us, that “men, in general, are people who do not want to do HIV tests and at times they do not want to use condoms; when you tell a man it’s time to use a condom they don’t want to understand” (71 y, m).

The CLs described a community space where stigma is still very present and that there are members who “will reduce their interaction with the person [living with HIV]” (49 y, m). Talking about stigma inside the community, the CLs mainly referred to experienced stigma – defined as stigmatising actions which do not align with international human rights (UNAIDS, 2020) – and anticipated stigma – defined as the expectation of being stigmatised by others due to a certain condition or behaviour (UNAIDS, 2020).

The CLs explained that people are “still scared to come out” (63 y, f) because the community understands HIV as a “sexual virus” (71 y, m) linked to “having sex with prostitutes” (71 y, m) and immoral sexual behaviour. The CLs felt that the anticipated and experienced stigma will also prevent their members from using PrEP. One female CL (31 y) explained, that “most of them are happy about it [PrEP] but the problem is they say it needs not to be seen that they take PrEP because the community will think otherwise of them”.

As well as describing the space as stigmatising, two of the interviewed CLs had stigmatising beliefs themselves. One CL (61 y, m) recommended people living with HIV to be given a mark because “you see someone to be beautiful because they have no mark to distinguish them”. The other CL (66 y, m) explained that people living with HIV should not be allowed to marry an HIV-negative person. He felt that this “will encourage people to change their moral behavior knowing that in the end it may happen that I may fail to marry the one I love because I have been found to be HIV positive” (66 y, m). However, few CLs felt that stigma “is not common because here at Mhlangatane HIV is widespread” (37 y, f). They thought that community members can talk openly about HIV and their status and “stigmatization is now coming to an end” (66 y, f). CLs emphasised the danger emerging from the messages sent by some leaders to the community.

CLs saw the dissemination of stigmatising and wrong messages through religious leaders as a major challenge and running in conflict with the dissemination of scientific information and the promotion of PrEP and HIV prevention. CLs explained that some churches disseminate the information that as a good Christian you cannot get infected with HIV, and hence, no medical treatment or prevention is needed because “God will take care of their health” (71 y, m). Therefore, the symbolism of being in church sends a message to the community that you do not need to use any prevention or treatment because “if you are in church then you are ok” (49 y, m). The CLs complained that many religious leaders do not include HIV prevention in their information programs. They only preach the word of God and do not “even take 20 minutes to preach about HIV” (39 y, m) during their services. The CLs felt that evidence-based information about HIV and HIV prevention should be regularly shared in the religious space.

### Message Content: What Are the Messages?

Most of the CLs regularly talk about HIV with their communities and emphasise that “people should check, prevent and know their HIV status” (33 y, m). The content of their messages mostly relates to “that people should protect themselves” (63 y, m) and that there are multiple options for HIV prevention. CLs emphasise the importance of condoms and that members should “take the condoms and put them in your pocket” (39 y, m) because members “must continue to use the condom” (63 y, m). CLs talk about the dangers of multiple concurrent partnerships and tell members “to avoid sleeping around, especially when you now know you are taking the pills but try and control yourself” (37 y, f). One CL (71 y, m) explained that promiscuity is an important value to some men “to prove that we are real men calling ourselves *Inganwa* [loosely translated meaning a man with so many women in a sexual relationship with] and also to boost our egos”.

The CLs also encourage their members to regularly test for HIV and counsel them on the importance of correct medication (ART) use. One CL reported that he “counseled her [sick community member] on the importance of taking treatment and asked if she would like me to take her for a test at the hospital” (71 y, m). Some CLs also talk to their members about the risk of the “consumption of alcohol” (39 y, m) and to “end up doing things [unprotected sex] that they are not supposed to do because they are drunk” (39 y, m).

Two CLs explained that their messages also include content that is designed to contest stigma and empower people who are being stigmatised, encouraging them to “not pay attention to what another person has to say” (48 y, f) about her or his health condition. If talking to someone living with HIV, they emphasise “not to be embarrassed of being HIV positive because many people are HIV positive and they are living happy lives” (71 y, m).

The CLs that had received some information about PrEP were happy to talk about it and advise them to “not to waste time and to go to the clinic to take PrEP” (37 y, f) and want “to give them the information” (66 y, f). A CL told us that she advised a member that there “is a PrEP at the clinic and it will help you, so that you can stop being worried all the time about HIV issues. Like now you will be rushing for HIV tests all the time, yet the pills will help protect yourself from getting infected with HIV. She [the member] was happy about that and wondered why I took so long to tell her of such a thing” (31 y, f).

### Message Delivery: What Are the Approaches?

One-on-one, couple, family, small group, male and female only, community, and church group meetings that take place within the home and at appropriate community locations are the key approaches to reach the community with messages about HIV prevention and PrEP.

CLs explained that they are a constant and easily accessible service for people within their community. They said that they have access to, and communicate with, the community members on a private, interpersonal level that others do not, in spaces that are comfortable and often intimate, such as in the CLs’ or a persons’ home. CLs explained that they have the trust and can access homes which allows them to build a deep understanding of the aspects of their family life that may have a big impact on the health behaviour. One CL explained that “if you have gone to their homes, they will tell you things that you do not know and be shocked” (40 y, f). Some members are afraid to go to the hospital and want to discuss private matters in their homes because “at home, they can talk and say it is like this and that” (40 y, f).

The CLs explained that community members seek them for direct advice, normally male to male, female to female, and highlight the potential of these interpersonal conversations. If a female CL talks to men, they would “discriminate against women” (37 y, f) saying that “I [female CL] do not know what I am talking about” (37 y, f). However, “a man could talk like this situation happened to them and they know it and seen it then in that way the men can be able to listen” (37 y, f). One-on-one discussions were also thought to be the most frequent approach used by traditional healers to communicate health messages, and therefore, their approach replicated what is socially acceptable and expected. The CLs felt that talking one-on-one to their members (with training) would show that they give enough time and can build enough rapport to show that PrEP “is a good pill that can help” (49 y, m) and motivate the member to visit the clinic for further information.

CLs said they also talk to the community in larger groups about HIV prevention and PrEP. These meetings take place in public settings where people usually meet, such as dip tanks (animal vaccination points where men frequently meet), the soccer field, in school, or at church. For men, who “don’t want to go to the hospitals” (33 y, m), those public community locations – as well as their homes – are important places to inform about HIV. One CL told us that together with a health NGO, “we visit their homes and encourage them to meet as men in their chiefdoms and teach them” (33 y, m) about HIV. The CLs said that men must be engaged within the chiefdom “because the men go there and they want to be educated” (39 y, m).

Although CLs said some community members and religious leaders did not think church was the appropriate space for the delivery of HIV prevention information, many CLs highlighted religious events as effective approaches to quickly disseminate information to many people because “we get most people in churches” (35 y, m). The CLs, some of them pastors themselves, explained that it is easy to talk about HIV issues in church because people consider the church as a reliable and credible platform to disseminate information and increase acceptance. This is highlighted by one CL, stating that “if we can get out and go to the Tinkhundla, chiefdoms, and the churches, then there is an impact that we can play” (49 y, m). CLs said that they do not mention clinics when initially giving information on PrEP. A CL explained that clinics are not the best fitting for every community member because “men are not being empowered [for HIV], they are only being blamed” (41 y, m). However, health workers were invited and often spoke at their community meetings.

### Message Persuasion: How Do They Persuade?

The CLs explained that they are able to persuade their community via trust, relatability, familiarity, empowerment, and shared faith. CLs said that the community trusts in their elected CLs and “do listen to me [CL] now as a leader” (71 y, m). The CLs felt that the community follows their recommendations and asks for their leader’s advice and support regarding HIV prevention because the members “have put their trust in me” (33 y, m) and if “they have a problem, they come to me [CL]” (33 y, m). The CLs highlighted that they have a close connection to the men and the youth and regularly advise them on HIV prevention. For example, CLs told us that men are too embarrassed to buy condoms in shops, and therefore, some male CLs purchase condoms to share with the community as this is “the simplest way to bring the condom close to them” (35 y, m). The CLs told us that the community members “love visiting me [CL] in my house” (35 y, m) to collect condoms.

The CLs felt that they are important for men because they do not trust in health workers but are able to relate to them as important role models. They (men) “are very exemplary” (71 y, m) and would visit, for example, dip tanks, for information if the Chief himself is there. However, if the “Chief himself is a very cultural person who doesn’t believe getting help from the clinic” (71 y, m) is “another problem” (71 y, m).

CLs explained that as they are part of the community, community members identify themselves with their leaders and are familiar with them. Unlike medical professionals or those visiting the community from NGOs, CLs hold similar values and talk in a way that replicates the local dialect, use words that hold local significance, and can discuss, name, and debate the – for example – medicines that traditional healers provide. The CLs felt that, depending on age and gender, different key groups identify and connect with them in ways that other health providers or promoters do not. CLs said that it is easy to talk to “people that are used to me [CL]” (35 y, m). Moreover, CLs explain that they know the cultural challenges of talking to certain population groups and that they can adapt their programs to meet the doubts and expectations of their community. For example, “for elders, it is like an insult” (33 y, m) to talk about HIV prevention including sex and sexuality because it is considered a taboo. Elders “do not take it [PrEP information] up quickly” (49 y, f). CLs are also able to provide personal examples and, as some are HIV positive themselves, can share their story to detail the process and persuade community members to visit the hospital.

Some CLs that also worked in churches explained that community members who attend church service believe in God and the Bible, and therefore, they trust their religious leaders and the health information given by them. In church, “they [members] listen and it is easy for them to accept that the virus is there and that we should take the treatment” (37 y, f). One CL explained that it is “just the information of the pastor” (45 y, m) that makes people change their behaviour. CLs felt that the people who visit church services hold similar views to their religious leaders and congregation as they share the commonality that “we are both Christians” (49 y, f) and that this commonality leads to a mutual need to protect the community from HIV. Moreover, they explain that it is very important to congregation members to be seen as holy and good Christians because they identify as a “child of God” (35 y, m) and therefore follow the advice given by their religious leader. This Christian behaviour is directed and controlled by religious leaders, and CLs said the congregation follows the information and restrictions given by them. CLs felt that church members fear being excluded or stigmatised by their congregation if not behaving as required by their religious leaders and norms. CLs explained that religious leaders promoting PrEP would increase its acceptance and interest within church congregations. If not promoted by religious leaders, some members might say that “Jesus never used any pills so for me kneeling down and praying is enough. I don’t use pills” (29 y, m).

CLs said that some men prefer visiting traditional healers rather than going to clinics or hospitals because they trust the traditional healers more than health workers. Some men “believe in the herbs […] instead of going to the hospital” (39 y, m). The CLs stated that the persuasive impact of traditional healers and HIV prevention was not to be underestimated. Their ability to persuade community members to take herbs instead of medicine was significant and was described as a reason that “our culture will kill us” (39 y, m). CLs felt that understanding how these traditional healers could be harnessed to share information that was as accepted as their support would be essential for future HIV prevention programs.

## Discussion

Our findings indicate that CLs feel they are crucial social actors in sustainably disseminating information about HIV and PrEP in their communities. The CLs described the social and health behaviours of their community as being influenced by religious, individual, and traditional values. Anticipated and enacted stigma and stigmatising beliefs about HIV and sex are still present in the community and are thought to have a negative influence on the acceptance and use of HIV prevention methods. Some religious leaders and traditional healers share stigmatising information about HIV which is considered to have a negative impact on the communities’ health behaviour. CLs regularly talk to their members about HIV prevention and promote the use of condoms and PrEP. Community and religious spaces – as well as interpersonal communication – are viable channels used by the different types of leaders to reach their community. The interviewed CLs are highly motivated to be actively involved in PrEP promotion.

Our work resonates with other studies from Eswatini and sub-Saharan Africa that recommend to actively involve CLs in PrEP programs (Bärnighausen et al., 2020; Berner-Rodoreda et al., 2020; Cowan et al., 2016; Nakasone et al., 2020) and that the interviewed CLs are positive towards PrEP and motivated to support its promotion in the community (Fadane, 2019). Furthermore, local leaders have been shown to play an important role in identifying and overcoming barriers for HIV prevention and PrEP uptake related to socio-cultural norms (Geldsetzer et al., 2020; Nannozi et al., 2017; Tarkang et al., 2018) and in influencing their communities HIV prevention behaviour (Chimatiro et al., 2020; Marashe, 2014). However, CLs’ perspectives about health programs are frequently overlooked and their potential is underestimated (Chimatiro et al., 2020; Young et al., 2021). CLs described that they were not involved in the PrEP demonstration project despite PrEP clients, health workers, and PrEP stakeholders repeatedly recommended to talk to CLs (Bärnighausen et al., 2020).

The messages CLs deliver are clear and are in line with the prevention options recommended by UNAIDS (UNAIDS, 2021b), consisting of the promotion and use of condoms, PrEP (by those that knew of it), one-partner relationships, and testing and adherence to ART. In addition to their constant physical availability within the community, the platforms CLs use to provide information were also clearly defined, with one-on-one interpersonal, couple, family, and community group meetings serving as appropriate depending on the content of the messages being shared. As with work from Uganda (Starmann et al., 2018), and communication theory (Rogers, 1995, 2002), our CLs believed interpersonal conversations are the most beneficial for changing people’s behaviour, as they have better opportunities to influence and empower community members to ask questions about PrEP without being ashamed of others reactions (Stankevitz et al., 2019). As with work by Berner-Rodoreda (2020), the community and group approaches CLs use to reach men are particularly important, because of the dynamic in conversation that can be built within a group of men when a ‘leader’ is present, as well as reaching those that do not access the regular health system due to fear, time, and transport issues (Berner-Rodoreda et al., 2020). CLs defined community spaces as those areas with enough space for many people such as soccer fields, dip tanks, and church grounds.

While the messages and platforms are clear, the influence of context on the persuasive ability of the CLs is more complex. CLs see that religious values are combined with tensions and contradictions between culture and HIV which is commonplace within the literature (Ochillo et al., 2017). HIV prevention in Eswatini can only be understood when considering the colonial history, the global political economy of HIV funding and activism, and the unique epidemiology of HIV and culture, religion, local traditions, and discourses (Root, 2014). Eswatini is a country where much of a generation was lost to AIDS, taking with it customs, practices, and norms, at a time when the political landscape was changing and much of the post-colonial African region was ideologically opposed to acceptance of support from ‘indirect rule’ (Prinsloo, 2007). Given the rapid incidence rise in Eswatini in the late 1980s and throughout the 1990s (Mabuza & Dlamini, 2017), external support from foreign agencies was accepted, but this support modelled much of the preferred development agendas and ‘decentralized despotism’ (Prinsloo, 2007). HIV and AIDS support from NGOs was not sustainable (Seckinelgin, 2005), and policy and research focus moved disproportionately towards sexual behaviour (Stillwaggon & Sawers, 2019), ignoring many of the contexts in which HIV flourished (Sastry & Dutta, 2011). On the contrary, CLs fight this persistent colonial legacy via their symbolism of the local and embody all that is Eswatini. In the words of Prinsloo (2007, p. 77), the identity that CLs symbolise is “*discursively produced, deployed and sustained*” in opposition to governance or – in our case – health programs that come from ‘outside’ (Prinsloo, 2007).

CLs are in a unique position to mediate information via this trusted and shared identity so that it is appropriately reproduced and received, and hopefully, acted upon. While our CLs make no direct reference to ‘discursively produced identities’ (Prinsloo, 2007) contributing to what we organised as ‘message persuasion’, the trust relatability, familiarity, and shared faith CLs describe the community as having in them is key to their access, but is also what enables traditional healers to continue to ‘treat’ HIV within these settings.

This need for a sustained identity within role models may also be of particular significance because of the number of orphans in the country (UNAIDS Country Factsheet Eswatini 2021, 2021). Before the roll-out of ART, 120,000 children were orphaned by AIDS and raised by grandparents and other carers (Makadzange & Dolamo, 2011). Losing those respected elders and parents who pass down information and convey constructions about identity and socio-cultural processes makes those social actors who deliver messages of significance important to be recognised as trustworthy and in the community’s best interest.

Applying this to practice via CLs means developing culturally sensitive PrEP communication frameworks tailored to the local context of the target group (Govender & Abdool Karim, 2018; Govender, 2021). Collaborating with health workers, researchers, and other PrEP stakeholders, CLs with a similar cultural background as the community can contribute to develop a context-sensitive communication, informed by a cultural competence framework (Campinha-Bacote, 2002; Govender, 2021), that is, a framework to develop health programs, tailored to a specific cultural context by focusing on “*cultural awareness, cultural knowledge, cultural skill, cultural encounters, and cultural desire*” (Campinha-Bacote, 2002: p. 181). Within this framework (Campinha-Bacote, 2002), CLs and health providers are given the tools to shape the community space in a way that can educate positively about sexuality, PrEP use, and other HIV prevention methods, without fearing stigmatising reactions or censuring. Moreover, they can actively approach members and offer individual advice, tailored to his or her personal situation and background.

Our CLs also spoke of stigmatising messages sent by religious leaders, traditional healers, and also some CLs, which continue to challenge the promotion of PrEP (Calabrese, 2020) and need to be overcome to increase community acceptance (Irungu & Baeten, 2020). Homesteads have previously been described as the most stigmatising environments in Eswatini (Root, 2010), and felt stigma in rural communities is attributed to lack of care seeking, social support, and poor treatment adherence (Root, 2010). Much work suggests that addressing stigma requires multidisciplinary approaches that are educational and local-specific (Kanduza, 2003; Root, 2010). Based on our findings, CLs could identify sources where stigma emerges, and react by engaging in one-on-one conversations where stories, experiences, and local narratives can be conveyed, alongside evidence-based information.

Evidence from Southern Africa points to a new framing of PrEP messaging to overcome such stigma and thereby can be empowering (Morton et al., 2020; Rivet Amico & Bekker, 2019), positive, and health-centred (Rivet Amico & Bekker, 2019). While continuing to discuss safe sexual practices is important, PrEP use in its whole does not have to relate to one specific (stigmatising) dimension of sexual behaviour, but rather a lifestyle choice that is protective and future-proofing (Bärnighausen et al., 2019; Rivet Amico & Bekker, 2019). If CLs can bring these messages into their intimate, trusted spaces, such as the homestead – where they feel they have the most impact – it could support broader approaches of PrEP demand creation campaigns and communication strategies that have only been minimally explored.

Studies from Zambia and Ethiopia have shown that workshops about evidence-based information on HIV prevention for religious leaders have helped to correct wrong information about HIV prevention and to overcome stigmatising beliefs (Feiruz Surur & Mirgissa Kaba, 2000; Wiginton et al., 2019). We argue that educating and involving local leaders can not only reduce the spread of stigmatising messages but also increase community awareness and PrEP acceptance in general.

Applying our findings by actively involving local leaders in PrEP promotion requires investment in training to inform local leaders about PrEP and reinforce existing knowledge about HIV prevention. Training should also involve communication approaches and strategies to harness the positive dimensions of the conflict we see within the HIV prevention communication space. An example of existing training in this field is the SAVE toolkit, developed by the International Network of Religious Leaders Living with or Personally Affected by HIV and AIDS (INERELA+) that equips religious leaders with evidence-based information on HIV and how to use this information appropriately in a religious setting to create awareness in the community (Church of Sweden INERELA+, n.d.). Similar toolkits can be developed to reflect the working context of CLs and traditional healers to prepare them to be involved in PrEP programs.

Moreover, we recommend offering training on developing and using positive messaging about PrEP (Stankevitz et al., 2019) in a way that harnesses the existing approaches of the leaders. Positive messages about PrEP should empower members to use HIV prevention methods and avoid fear- and stigma-based messages about HIV (Stankevitz et al., 2019).

### Limitations

We acknowledge that our findings represent a relatively small sample size and are limited to the experiences of CLs and do not capture the perspectives of other key community figures such as religious leaders and traditional healers (Audet, Clemens, et al., 2020; Audet, Pettapiece-Phillips, & Salato, 2020; Ransome et al., 2018). PrEP stakeholders (Bärnighausen et al., 2020) and health workers (Barnighausen et al., 2020) have already highlighted the need to involve CLs in PrEP promotion programs in Eswatini. However, community members’ perspectives need to be explored to understand the credibility and validity of these recommendations and to learn about potential other community actors that can play an important role in community PrEP roll-out. The access that CLs have to the community via the unique dual (traditional and constitutional) government system in Eswatini (Government of Eswatini, 2021) – where CLs play an important role in various decision-making processes (Dlamini, 2013) – might not be transferable to settings where this model does not exist.

Although we built space into our data collection instruments for reflexive comments, as well as into our debriefings and then data analysis process, at no point did we acknowledge that the voices and stories of traditional healers were not included in our work. This may be because of an unconscious bias that traditional healers are not relevant or that their reach in the community is not as extensive as CLs. However, our research shows that traditional healers may be particularly useful in understanding the way traditional healing is perceived versus medicine prescribed in a clinic. Based on this research with CLs, we have conducted further interviews with religious leaders in Eswatini and will conduct another round of data collection with traditional healers.

Further research is required to understand where, why, and how stigmatising messages can arise in communities and how they influence community members’ attitudes and behaviour. Moreover, CLs were not part of the initial study population and the interviews were conducted after PrEP had been made available to the general population. Ensuring CLs are involved before implementation could be essential for the success of future interventions.

## Conclusion

CLs in Eswatini feel that they and other local leaders can play a significant role in the support of PrEP uptake and use. CLs emphasised that they would like to see a clear health-sector strategy for their involvement in future PrEP roll-out, even though they felt confident about their general HIV knowledge and about using their communication and community engagement methods and spaces for discussions of HIV and PrEP. They would benefit from general education about PrEP and specific education regarding the places and times when PrEP becomes available in their communities and more concrete ideas on how they could best refer potential PrEP clients to health facilities and encourage PrEP use. Helping CLs positively frame PrEP and HIV prevention messaging may overcome stigmatising messages. Understanding how to better leverage trust in CLs for information dissemination will be pivotal for the success of future HIV prevention programs.

More knowledge is required regarding the role of other influential local leaders – such as religious leaders and traditional healers – and community members to ensure consistent and non-stigmatising messaging reaches those who want to use PrEP and could benefit most.
